# *Ex vivo* assays to predict enhanced chemosensitization by hyperthermia in urothelial cancer of the bladder

**DOI:** 10.1371/journal.pone.0209101

**Published:** 2018-12-14

**Authors:** Nathalie van den Tempel, Kishan A. T. Naipal, Anja Raams, Dik C. van Gent, Martine Franckena, Joost L. Boormans, Roland Kanaar

**Affiliations:** 1 Department of Molecular Genetics, Erasmus University Medical Center, Rotterdam, The Netherlands; 2 Department of Molecular Genetics, Oncode Institute, Erasmus University Medical Center, Rotterdam, The Netherlands; 3 Department of Radiation Oncology, Erasmus MC Cancer Institute, Rotterdam, The Netherlands; 4 Department of Urology, Erasmus MC Cancer Institute, Rotterdam, The Netherlands; Columbia University, UNITED STATES

## Abstract

**Introduction:**

Bladder cancer (urothelial carcinoma) is a common malignancy characterized by high recurrence rates and intense clinical follow-up, indicating the necessity for more effective therapies. Current treatment regimens include intra-vesical administration of mitomycin C (MMC) for non-muscle invasive disease and systemic cisplatin for muscle-invasive or metastatic disease. Hyperthermia, heating a tumor to 40–44°C, enhances the efficacy of these chemotherapeutics by various modes of action, one of which is inhibition of DNA repair via homologous recombination. Here, we explore whether *ex vivo* assays on freshly obtained bladder tumors can be applied to predict the response towards hyperthermia.

**Material and methods:**

The cytochrome C release assay (apoptosis) and the RAD51 focus formation assay (DNA repair) were first established in the bladder cancer cell lines RT112 and T24 as measurements for hyperthermia efficiency, and subsequently tested in freshly obtained bladder tumors (n = 59).

**Results:**

Hyperthermia significantly increased the fraction of apoptotic cells after cisplatin or MMC treatment in both RT112 and T24 cells and in most of the bladder tumors (8/10). The RAD51 focus formation assay detected both morphological and numerical changes of RAD51 foci upon hyperthermia in the RT112 and T24 cell lines. In 64% of 37 analyzed primary bladder tumor samples, hyperthermia induced similar morphological changes in RAD51 foci.

**Conclusion:**

The cytochrome C assay and the RAD51 focus formation assay are both feasible on freshly obtained bladder tumors, and could serve to predict the efficacy of hyperthermia together with cytotoxic agents, such as MMC or cisplatin.

## Introduction

Bladder cancer is the fifth most common malignancy in Europe, accounting for 150,000 cases per year [1]. Bladder tumors are associated with specific clinical problems that are highly distressing for patients and, in monetary terms, make bladder cancer the most expensive malignancy per treated patient [2]. The disease presents itself as either non-muscle invasive bladder cancer (NMIBC, 75%) or muscle-invasive bladder cancer (MIBC, 25%) [3]. Although the 5-year survival rate of NMIBC patients is more than 90%, as much as 50% of all NMIBC patients will present with cancer recurrence after transurethral resection (TUR) of the tumor. Therefore these patients require thorough follow-up and adjuvant intravesical therapies [3]. One of the major clinical challenges is to improve the efficacy of adjuvant therapies to reduce the risk of recurrences, thereby minimizing patient burden and costs. The standard treatment of non-metastatic MIBC is neo-adjuvant chemotherapy and radical surgery, which is associated with high morbidity rates and even mortality [4]. Nonetheless, the 5-year survival rate of patients with MIBC is a mere 55% and this has not improved over the last decades [4,5]. All these clinical traits of bladder cancer clearly illustrate the necessity to find new, more effective and less burdensome therapies [6–8].

One of the most widely used approaches to reduce the risk of recurrence and progression of NMIBC cancer is intra-vesical treatment, which involves rinsing the bladder with a therapeutic compound. A commonly used compound to reduce the risk of recurrence in intermediate-risk NMIBC is mitomycin C (MMC) [9]. Systemic administration with cisplatin is the most preferred drug for MIBC in the neoadjuvant, adjuvant or palliative setting. Interestingly the efficacy of both MMC and cisplatin increases upon addition of hyperthermia, during which the tumor is heated to 40–44°C [10]. Hyperthermia has therefore attracted much attention to complement current bladder cancer treatment modalities. Adding hyperthermia to intra-vesical MMC treatment has previously demonstrated to improve its efficacy [8,11,12] and recently a randomized trial showed similar efficacy for intravesical chemohyperthermia compared to instillations with *Bacillus Calmette Guérin* in intermediate-risk NMIBC [13].

Hyperthermia has a plethora of biological effects on cells that cause cells to be more sensitive to the cross-linking agents MMC and cisplatin [14,15]. For instance, hyperthermia enhances blood flow and increases vessel permeability, which causes more of the chemotherapeutic drug to be delivered to the tumor [10]. Cisplatin uptake is enhanced in the cell by hyperthermia-mediated alteration of cellular membrane properties: it becomes more fluid [16], and the copper transporter CTR1, responsible for transport of cisplatin into the cell, is upregulated [17]. Moreover, in the specific case of the bladder wall, it has been suggested that hyperthermia might aid in overcoming its impermeable structure [18]. Thus, hyperthermia helps to overcome drug transport barriers of bladder tumors. However, once the drug has penetrated into the cells, hyperthermia has other means to increase cisplatin and MMC efficacy, namely by inhibiting DNA repair [19,20].

Both cisplatin and MMC are DNA crosslinking agents that can cause inter-strand crosslinks (ICLs). These lesions are extremely toxic for cells and their restoration requires a tightly organized combination of DNA repair pathways [21]. The major ICL repair pathway produces a double strand break (DSB), which requires repair via homologous recombination (HR) [22–24]. HR uses the information from an intact copy of the broken DNA to faithfully restore the break. This step requires strand invasion, during which the broken DNA infiltrates the intact sister chromatid, a process orchestrated by the recombinase RAD51 [22,25]. RAD51 localization to DNA breaks is aided by BRCA2 [26], a protein which is specifically degraded by hyperthermia [19]. By degrading BRCA2, hyperthermia inhibits HR and thereby a late step of DNA repair for damage inflicted by the ICL-inducing agents MMC and cisplatin.

Here, we explore whether hyperthermia-mediated inhibition of HR can be used as a marker for increased treatment efficacy in bladder cancer. We use recently developed *ex vivo* assays on fresh bladder tumor biopsies, which allow testing of potential treatments prior to administering them to patients [27]. We employ these assays to (1) determine hyperthermia’s potentiation of MMC and cisplatin treatment of bladder tumor cells, using a cytochrome C-release assay, and (2) determine the ability of the cells to form RAD51 foci after hyperthermia as a potential biomarker for efficacy of hyperthermia treatment [28,29].

## Material and methods

### Experimental outline and treatment timing

To explore the effects of hyperthermia on HR and cell killing, the bladder cancer cell lines (RT112 and T24) and freshly obtained bladder tumors were subjected to hyperthermia and various anti-cancer treatments. Hyperthermia was given in an incubator set at 42°C with a controlled environment (5% CO_2_ and 20% O_2_), while the control was left in the 37°C incubator. Cell cultures were placed in the hyperthermia incubator for 75 minutes, allowing 15 minutes for the medium to reach desired temperature and 60 minutes of effective hyperthermia. If cells or tissues were irradiated, they received the indicated dose from a cesium-137 source at 0.64 Gy/min, directly following hyperthermia. When cells were treated with the radiomimetic agent Zeocin (0.5 mg/ml, InvivoGen), MMC (Sigma-Aldrich) or cisplatin (Accord Healthcare Limited), the dose was added directly before hyperthermia. Two hours after addition of the chemotherapeutic, the cells were washed once with PBS and the medium was refreshed.

### Sample collection

Bladder tumors (n = 59) were collected at Erasmus Cancer Institute Rotterdam, the Netherlands between 2012 and 2016 (**S1 Table)**. Eight of the collected tumors (B125, B127, B133, B151, B155, B162, B163 and B166) contained no tumor cells, and were therefore excluded from analysis. All other samples were urothelial carcinomas obtained by Transurethral Resection (TUR). The tumors were collected as surgical residual material under the “Code Proper Secondary Use of Human Tissue”, founded by the Dutch Federation of Medical Societies (www.fmwv.nl). Patients could choose not to participate by means of an opt-out system. Patient data was blinded for the researchers by unique coding, so that individual data could not be traced back to the patients. This study was approved by the Medical Ethical Committee of the Erasmus Medical Center in Rotterdam, the Netherlands (MEC-2012-113).

### Sample handling

Directly after TUR the biopsies were transported to the laboratory in RPMI-1640, 10% fetal calf serum and 1% Penicillin/Streptomycin. Upon arrival in the laboratory, the sample was examined macroscopically and was either left whole or subjected to dissociation. Whole tissue material was manually cut into smaller pieces (maximally 3 mm) that allowed it to stay alive *ex vivo* for the duration of the RAD51 focus formation assay (~four hours). After performing an experiment, the intact material was formalin fixed overnight and subsequently paraffin embedded (FFPE). Tumors were dissociated using the gentleMACS Dissociator and the corresponding protocol (Miltenyi Biotec). The resulting cell suspension was subsequently cultured on coverslips in Amniomax C-100 medium (Gibco Life Technologies) as previously described [27]. Both the RAD51 focus formation assay and cytochrome C release assay were performed on the dissociated tumors between 24–48 hours culturing. After an experiment, cells were fixed in 4% PFA for 15 minutes.

### Cell culture

Both RT112 and T24 were maintained in 1:1 DMEM/F10 culture medium, supplemented with 10% FCS and 1% Penicillin/Streptomycin in an incubator set at 37°C, 20% oxygen and 5% CO_2_. Cells were kindly provided by dr. Ellen Zwarthoff and regularly tested for mycoplasma and distinguished on morphology.

### Sample preparation and immunoblotting

RT112 and T24 cells were exposed to hyperthermia and lysed directly afterwards in Laemmli sample buffer (2% SDS, 10% Glycerol and 60 mM Tris pH 6.8 in PBS) by heating at 95°C for 5 minutes. The sample was passed through a syringe to reduce viscosity. Protein concentration was determined by the Lowry protein assay [30], before 50 μg protein was prepared with loading buffer (0.01% bromophenol blue and 0.5% β-mercaptoethanol). Samples were then separated on a 5% SDS-PAGE gel and transferred to a PVDF membrane. Proteins were detected with primary antibodies mouse-anti-BRCA2 (OP95, Calbiochem, 1:1000) or mouse-anti-PARP-1 (Alexa 1:1000) and HRP-conjugated Sheep anti-mouse IgG (H+L) (1:2000, Jackson ImmunoResearch) as secondary antibody. Prior to signal detection on an Alliance 4.7 (Uvitec Cambridge), membranes were incubated with ECL substrate (1:1 mixture of A: 0.1M Tris-HCl pH 8.5, 2.5 mM Luminol, 0.4 mM p-Coumaric acid and B: 0.1 M Tris-HCl pH 8.5, 0.02% hydrogen peroxide).

### RAD51 focus formation assay

The RAD51 focus formation assay on intact bladder tissue was performed as described previously [28]. Briefly, after hyperthermia treatment, samples were irradiated with 5 Gy and fixed two hours after irradiation ended. After FFPE-treatment, the samples were sliced in 4 μm thick sections, and prepared for immunofluorescent labelling with primary antibodies mouse-anti-RAD51 (1:200, GeneTex clone14B4 GTX70230) and rabbit-anti-GMNN (1:400, Proteintech Group 10802-1-AP), and secondary antibodies Alexa Fluor goat-anti-rabbit 594 and goat-anti-mouse 488 (ThermoFisher Scientific). The RAD51-assay performed on established cell lines RT112 and T24 and on attached cells from dissociated tumors, was slightly altered: (1) cells were fixed with 4% PFA for two hours after irradiation, unless stated otherwise in the Figs, and (2) the deparaffinization and target retrieval steps were omitted. S-phase cells in RT112 and T24 cell lines were detected by EdU instead of geminin. Cells were labelled with 10 μM EdU (Invitrogen) 75 minutes prior to fixation, which was detected using a Click-IT reaction according to manufacturer’s protocol (Invitrogen), prior to immunostaining of RAD51.

### Clonogenic survival

Clonogenic survival was performed using an “immediate plating after treatment” protocol [31]. In brief, a feeder layer of 20,000 primary fibroblasts irradiated with 40 Gy and 100,000 RT112 or T24 cells were seeded on separate 60 mm dishes. The next day, RT112 or T24 cells were subjected to indicated treatments, immediately trypsinized and seeded at appropriate densities. Colonies were allowed to grow for 10 days before being fixed and stained in 45% methanol (v/v), 45% dH2O (v/v) and 10% Acetic Acid (v/v) and 2.5% Coomassie Brilliant Blue (w/v).

### Cytochrome C release assay

Cells were treated with hyperthermia in combination with the indicated chemotherapy. After two hours, medium was refreshed and 1:1,000 of the apoptosis inhibitor Q-VD-OPh (20 μM, MP Biomedicals) was added for 24 hours, after which the cells were fixed with 4% PFA for 15 minutes. Fixed cells were subsequently labelled with mouse-anti-Cytochrome C (1:100, BD Pharmingen) and secondary antibody Alexa Fluor goat-anti-mouse 488 (ThermoFisher Scientific).

### Image acquisition and foci counting

All images for RAD51 foci analysis were maximum projection of Z-stacks with an increment of 1 μm, obtained with a Leica TCS SP5 confocal microscope with a 63x oil immersion (n.a. 1.4) objective. Image size was 1024 x 1024 pixels and 82 x 82 μm. Foci were quantified using a previously described home-made macro within FIJI (Image J1.50i [32]) [33] was used to quantify foci. Cells were selected based on the perimeter of EdU staining, and the MaxEntropy thresholding algorithm was prior to the ‘Analyze Particles’-function. Images for measurement of Cytochrome C-release were obtained with the same confocal microscope but with different settings: a 20x dry objective was used to obtain images sized 1024 x 1024 pixels and 247 x 247 μm. Cells were manually analyzed and counted using Z-stacks with an increment of 2 μm on 5–10 independent stacks from one sample.

### Statistics

All graphs were generated in GraphPad Prism 5.02. For the apoptosis assay, the proportion apoptotic cells, and a standard error of the proportion were determined by dividing the number of cytochrome C releasing cells by the total population. Statistical tests used to determine significance of the results are indicated in the text or figure legends.

## Results

### Differential sensitivity to chemotherapeutics in RT112 and T24 cell lines

**Fig 1:**
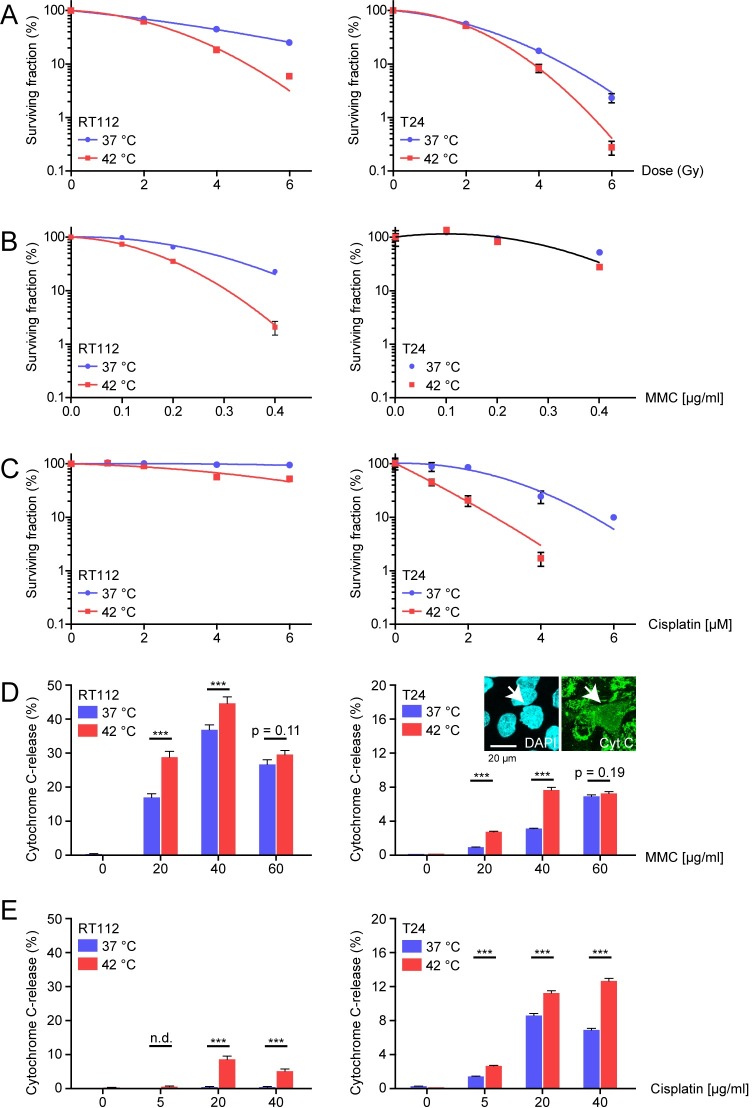
Survival responses of tumor cell lines to MMC and cisplatin. **A)-C)** Colony survival assays of bladder cancer cell lines RT112 and T24. Cells were treated without (blue) or with hyperthermia (red), and exposed to either **A)** irradiation up to 6 Gy, **B)** MMC up to 0.4 μg/ml and **C)** cisplatin up to 6 μM. **D)** and **E)** Graphs presenting percentages of cells with cytochrome C-release 24 hours after treatment with hyperthermia and indicated doses of **D)** MMC or **E)** Cisplatin. Cells were treated with caspase-inhibitor Q-VD-OPh to prevent full completion of the apoptosis-program. The arrow in the Fig embedded in panel D) T24 indicates a cell scored as Cytochrome C-release-positive. At least 500 cells were analyzed for each condition, bars represent proportion ± standard error of the proportion. Asterisk indicate significance as determine by Students’ *t-*tests: * *p*<0.05; ** 0.001<*p*<0.01; *** *p*<0.001. Exact *p-*values can be found in S2 Table.

With the goal to find preclinical markers which can predict whether hyperthermia is a useful addition to the treatment of bladder cancer, we started by establishing a functional survival assay suitable for freshly obtained bladder tumors. We first set up the assay in two established bladder cancer cell lines that have been used in hyperthermia research before: T24 and RT112 [34]. We determined the clonogenic survival capacity of heated or unheated RT112 and T24 cells treated with irradiation (Fig 1A), MMC (Fig 1B) and cisplatin (Fig 1C). We chose to treat cells with hyperthermia for 60 minutes at 42°C, corresponding to the time and temperature bladder cancer is treated using the BSD-1000 or BSD-2000 (BSD Medical, Salt Lake City, UT, USA) [8]. In both cell lines, this dose of hyperthermia sensitized cells to irradiation (Fig 1A), but there was clear difference in the sensitivity to both chemotherapeutics: T24 was relatively resistant to MMC treatment and could hardly be sensitized by hyperthermia, while RT112 was very sensitive to MMC, and this was further enhanced by hyperthermia (Fig 1B). This difference in MMC-sensitivity in these cell lines has previously been described, although tested with 43°C hyperthermia and by employing the MTT assay, which provides a measure of proliferation and does not test genetic viability [35]. For cisplatin we found the opposite of MMC sensitivity in RT112 and T24 cell lines (Fig 1C). The differential response towards cisplatin in unheated conditions in RT112 and T24 cells has been described before, and was attributed to a 2.5 fold-increased formation of ICLs in T24 relative to RT112, and increased levels of glutathione, a molecule associated with cisplatin-resistance, in RT112 [36,37].

To investigate chemotherapeutic sensitivity in tumors, an alternative approach to the colony survival assay is needed. Although bladder cancer tissue can be dissociated and cultured outside of the body, this can only be done temporarily, and therefore an alternative survival assay should be short-term [27]. We therefore selected an easily detectable, early step in the apoptosis pathway: release of mitochondrial cytochrome C [38]. During the assay, cells were exposed to selected treatments and subsequently treated with an apoptosis-inhibitor (Q-VD-OPh). The apoptosis inhibitor prevents cells from undergoing the full apoptotic program and therefore release of cytochrome C from the mitochondria can be quantified. After 24 hours, the cells were fixed and stained for cytochrome C, and the fraction undergoing release was determined (Fig 1D).

The cytochrome C-release assay was tested on RT112 and T24 cells, with or without hyperthermia, treated with increasing doses of MMC (Fig 1D) and cisplatin (Fig 1E). Importantly, hyperthermia itself did not induce cytochrome C-release (Fig 1D and 1E). MMC did induce cytochrome C release in both RT112 and T24 cells but, as in the colony formation assay, RT112 was more sensitive, as a much higher percentage of cells displayed MMC-induced cytochrome C release compared to T24. Interestingly, the cytochrome C release assay detected maximum treatment effects of MMC at 40 μg/ml. This is illustrated in RT112, where the number of apoptotic cells diminished when the concentration of MMC was increased from 40 to 60 μg/ml. Moreover, hyperthermia elevated the percentage of apoptotic cells at lower concentrations of MMC (20–40 μg/ml), but failed to do so at 60 μg/ml MMC in both cell lines (Fig 1D). It is possible that the high concentration of MMC induces necrosis, which is not inhibited by the apoptosis inhibitor and therefore not detected in the cytochrome C release assay. Corresponding to results from the colony survival assay, RT112 was relatively insensitive to cisplatin, while a significant amount of T24 did undergo apoptosis upon treatment (Fig 1E). In both cell lines, hyperthermia elevated the percentage of cells with released cytochrome C (Fig 1E).

### Cytochrome C in tumors

In both RT112 and T24, the cytochrome C release-assay could be used to quantitate their sensitivity towards cisplatin and MMC, and demonstrate their enhanced sensitivity upon additional hyperthermia treatment. We therefore continued by testing the suitability of the assay to detect sensitivity of dissociated tumors. Because the number of coverslips that could be obtained was usually small, we selected limited conditions to be tested: control, 40 μg/ml MMC and 40 μg/ml cisplatin. Because hyperthermia by itself did not induce apoptosis in RT112 and T24, only MMC and cisplatin treatment were combined with hyperthermia.

**Fig 2:**
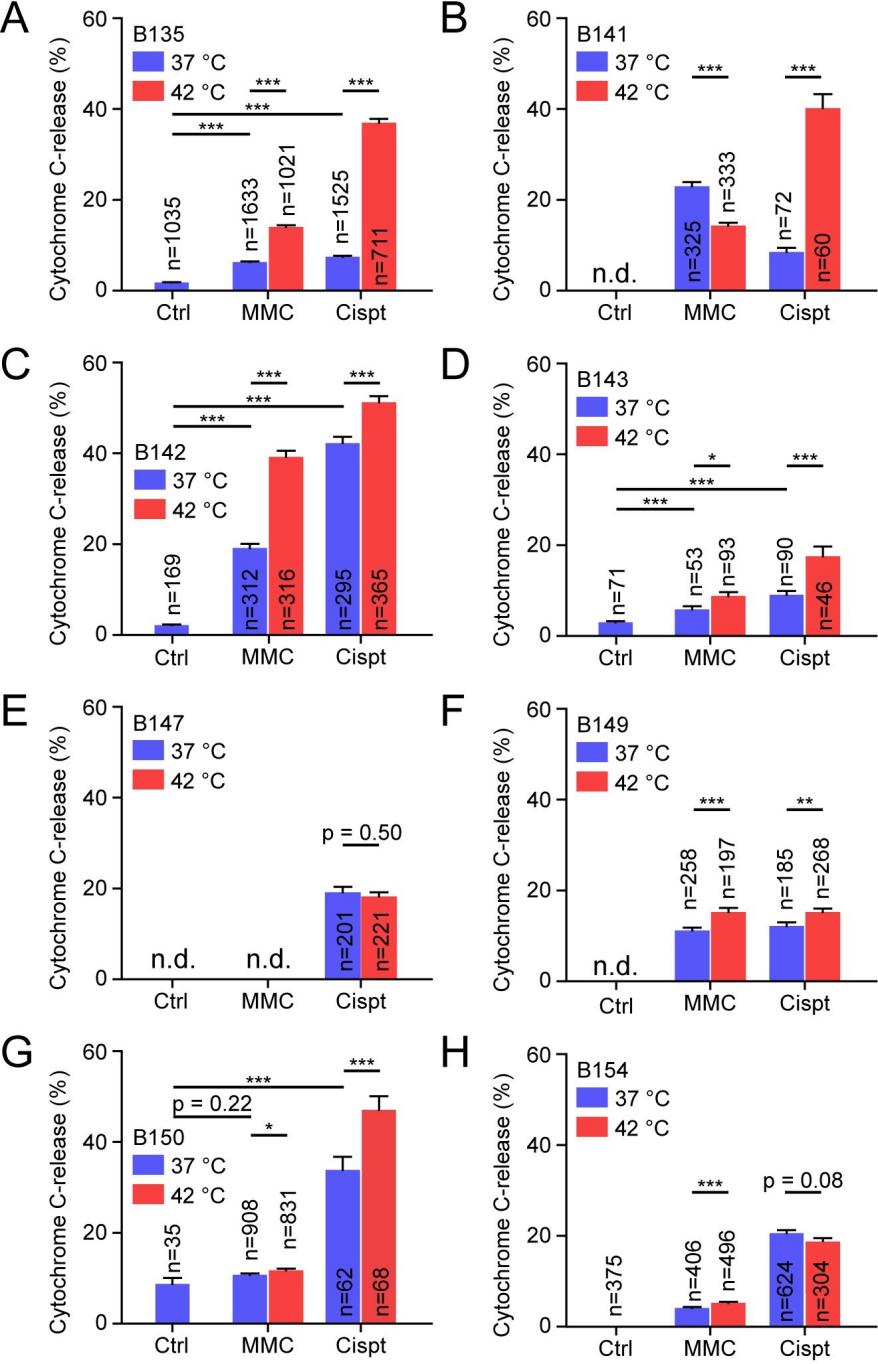
Cytochrome C release in dissociated bladder tumors. Graphs representing the percentage of Cytochrome-C-positive cells in dissociated bladder tumors. Tumor cells were cultured for 1–2 days before being subjected to hyperthermia in combination with 40 μg/ml MMC or 40 μg/ml cisplatin. After treatment, medium was refreshed and the caspase-inhibitor Q-VD-OPh was added. After 24 hours, cells were fixed, stained and scored for Cytochrome C-release positivity. **A)-H)** all represent different tumors. N.d. = not determined. The number of cells analyzed are indicated in each bar, which represent proportion ± standard error of the proportion. Asterisk indicate significance as determine by Students’ *t-*tests: * *p*<0.05; ** 0.001<*p*<0.01; *** *p*<0.001. Exact *p-*values can be found in S3 Table.

Out of 13 bladder tumors dedicated for the cytochrome C release-assay, eight tumors produced sufficient numbers of attached bladder cells to perform the assay (Fig 2A–2H). Because the size of bladder tumors obtained was limited, or because cells did not attach to coverslips, not all samples were tested for the full set of conditions. For the five tumors for which unheated control data was obtained (Fig 2A, 2C, 2D, 2G and 2H), the percentage of cells undergoing cytochrome C release increased upon MMC treatment in four samples (Fig 2A, 2C, 2D and 2H), as was the case in all cisplatin treated samples. Hyperthermia increased the percentage of apoptotic cells in six out of seven samples treated with MMC (Fig 2A, 2C, 2D, 2F, 2G and 2H) and decreased the percentage in one (Fig 2B). This decreased number of apoptotic cells upon hyperthermia treatment may again be due to an increase in necrosis rather than apoptosis. For cisplatin treatment, hyperthermia increased the percentage of apoptotic cells in six out of eight samples (Fig 2A, 2B, 2C, 2D, 2F and 2G) and did not alter the percentage in the remaining two (Fig 2E and 2H).

### Quantitative and morphological differences in RAD51 foci in T24 and RT112 lines

**Fig 3:**
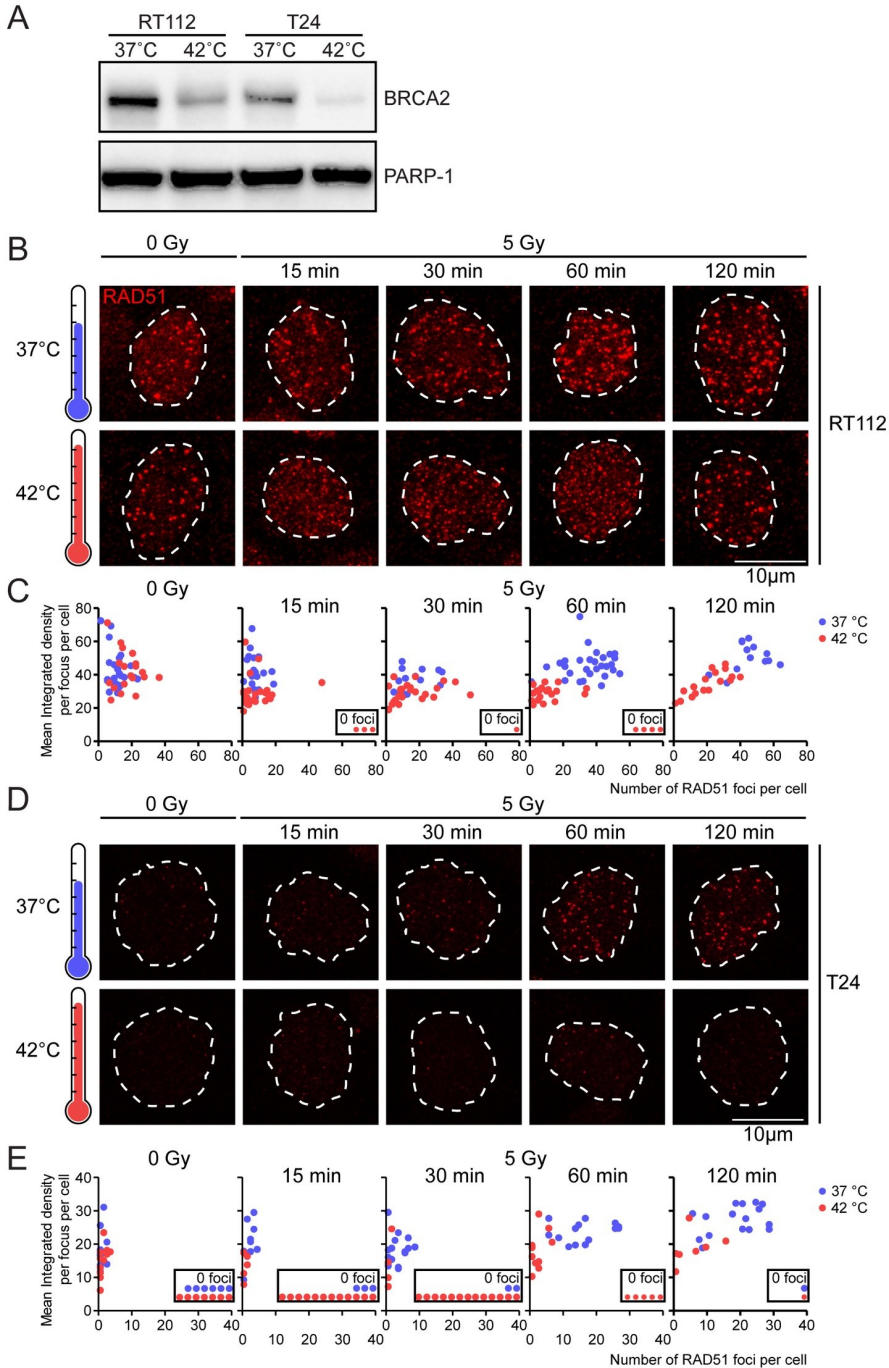
Hyperthermia induces distinct changes in RAD51 foci in RT112 and T24 cell lines. **A)** Immunoblots of samples treated with or without hyperthermia, probed for BRCA2 and PARP-1 as a loading control. **B)** RT112 cells were treated with or without hyperthermia and subsequently irradiated with the indicated dose. Cells were then incubated at 37°C, and fixed at indicated time-points afterward irradiation. EdU was present for one hour prior to fixation to identify S phase cells. Cells were stained for EdU and RAD51. Pictures are a maximum projection of a Z-stack, and the dotted lines denote the perimeter of EdU-positive nuclei. **C)** The 2-dimensional graph expresses a quantified classification of RAD51 foci. Each dot in the graph represents one cell, plotted based on the number of RAD51 foci in its nucleus (y axis) and their integrated density (x axis); a derivate of intensity and area per nucleus. Cells without foci are presented in the embedded figure noting ‘0 foci’. **D)** and **E)** As panel B and C, but for T24 cells.

The *ex vivo* RAD51 focus formation assay has been used successfully to determine the HR-status of freshly isolated mamma tumors [28,29]. To check whether this assay can be used to determine the effectivity of heat-induced HR-deficiency in bladder tumors, we first studied the appearance of RAD51-foci in the T24 and RT112 cell lines [34]. To measure hyperthermia-induced HR-deficiency in these cell lines, we first confirmed that BRCA2 was degraded upon hyperthermia in these cell lines (Fig 3A). The initial level of BRCA2 relative to the loading control PARP-1 was higher in RT112 than in T24, possibly because BRCA2 is mostly expressed in S-phase [39], in which relatively more RT112 cells than T24 cells reside [40]. However, the BRCA2-levels dropped substantially in both cell lines when hyperthermia was applied, implying that hyperthermia indeed affects HR in these two bladder cancer cell lines (Fig 3A).

Next, we analyzed the effects of hyperthermia treatment on the appearance of RAD51 foci at different time points after 5 Gy irradiation. Proper quantification of RAD51 focus formation capacity under different experimental conditions requires identification of S-phase cells[41]. Therefore, EdU was included in the culture media for an hour prior to fixing the cells, and only cells positive for EdU-staining were analyzed. Within the EdU-positive cells, the RAD51-foci were analyzed for both number per cell and integrated density, an expression of their intensity and area [33]. In the RT112 cell population, we found an average of 11 foci per nucleus when cells had not been irradiated (Fig 3B and 3C). When cells were irradiated, this number of foci increased to 35 foci at 60 minutes. Hyperthermia lowered the number of RAD51-foci and changed their morphology to less intense, smaller, and more dispersed at all time-intervals after irradiation. This change in their visual appearance (Fig 3B) was reflected in a reduction in their integrated density, as more objective measurement of their morphology (Fig 3C). The hyperthermia-induced reduction in foci number and change in appearance lasted at least until 60 minutes after irradiation (as indicated by the separation of the populations of red and blue dots in Fig 3B, lower panel, 60 min). At two hours after irradiation cells with an increased number of foci with a larger integrated density started to re-appear in the population (Fig 3C, 120 min). The RAD51-focus behavior observed in the T24 cell line was markedly different from the RT112 cell line: almost all unirradiated cells had no RAD51 foci, and they were induced only at a slow rate. The number of cells that did not have any foci greatly increased upon hyperthermia (Fig 3D and 3E). Moreover, the average number of foci in a hyperthermia-treated cell was much lower relative to 37°C controls (Fig 3D and 3E).

### *Ex vivo* RAD51 focus formation assay on fresh bladder tumor biopsies

The number and appearance of RAD51-foci altered substantially upon hyperthermia in both bladder cancer cell lines, indicating that RAD51-focus formation could be a suitable biomarker for determining the effectivity of hyperthermia in bladder tumors. Out of 57 collected tumor samples, 48 samples contained sufficient tumor material to perform a RAD51-focus formation assay. We tested both intact tissue and dissociated tumors that were cultured for 1–2 days, to test the robustness and versatility of the assay. RAD51 foci were induced using either irradiation (5 or 10 Gy) or the radiomimetic agent Zeocin [42]. After DNA damage induction the cells were incubated at 37°C for two hours before fixation to allow RAD51 foci to form. Fixed tissue or cells were stained for RAD51, while geminin (GMNN) was used as a marker for S- and G2-phase cells.

**Fig 4:**
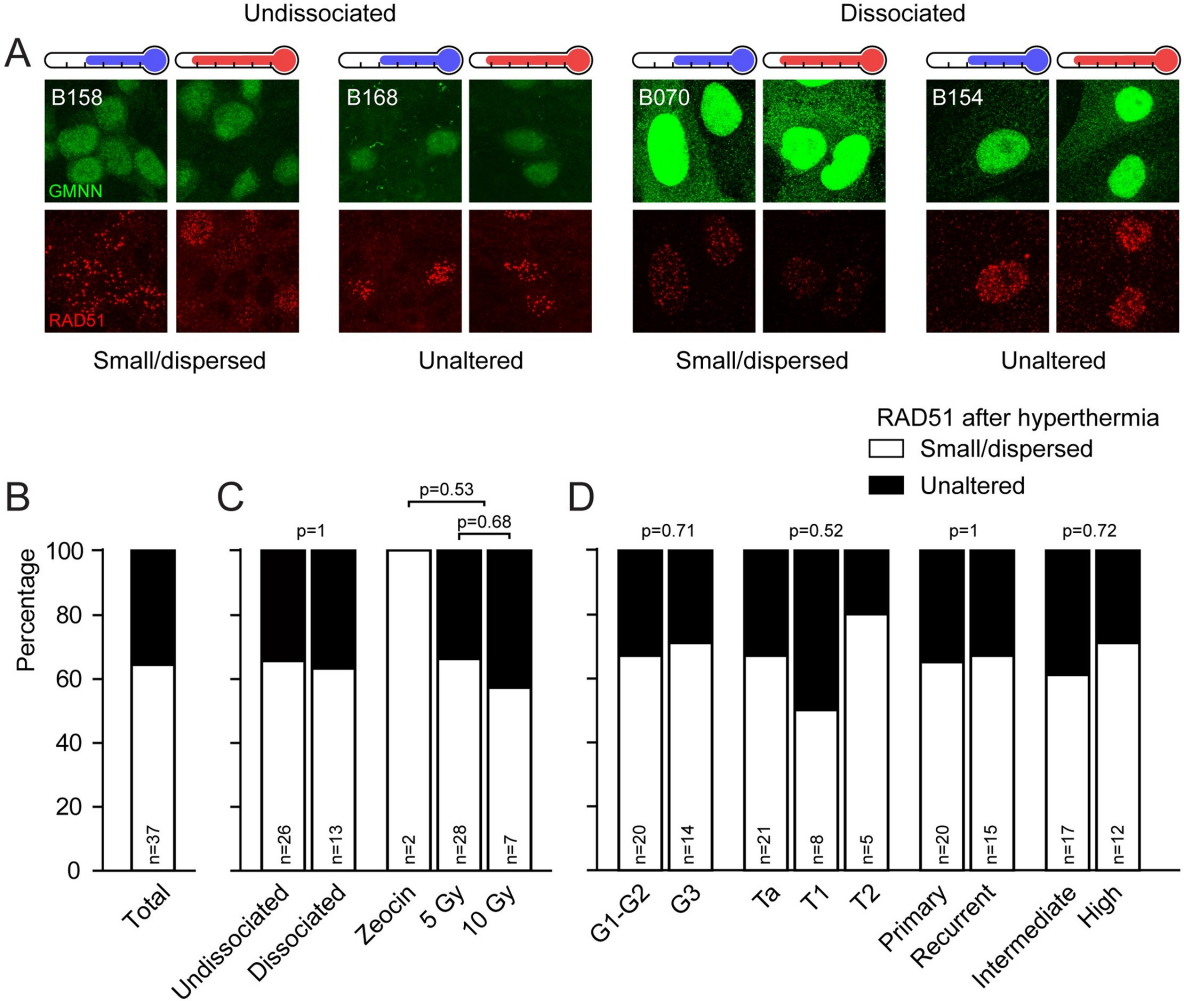
Classification of RAD51-focus morphology upon hyperthermia in human bladder cancer. **A)** Two hours after DNA-damage induction irradiation or zeocin, tumors were fixed and prepared for immunohistochemistry and subsequently stained for GMNN (green) and RAD51 (red). Pictures presented are examples of both undissociated and dissociated tumors exhibiting the two distinct morphological responses of RAD51-foci. Panels below the blue thermometer received no treatment before damage induction; panels below red thermometers were first treated with hyperthermia. The images are maximum projections of Z-stacks from representative areas of the tumors. **B)** Graph representing the frequency distribution of the morphological response of RAD51-focus upon hyperthermia treatment. The white part of the bar represents foci which have become smaller and more dispersed upon hyperthermia, the black part represents foci that did not change in morphology. Each bar contains the number (n) of analyzed tumors below. **C)** Frequency distribution based on the tumor preparation step (undissociated or dissociated) or on the method of damage induction. Two tumors are classified in both methods of tumor preparation, because both were performed on the same tumor, and had the same result. No differences were found in the foci-response distribution between the technical methods (Fisher’s Exact test, *p*-values stated in the Fig). **D)** Distribution of RAD51-foci response upon hyperthermia based on clinical data (available for 36 tumors). No differences were found in the frequency distribution based on tumor grade (G1 and G2 vs G3, Fisher’s exact test), tumor stage (Ta, T1 and T2, Chi-square), primary versus recurrent tumors (Fisher’s exact test), or in the EAU risk classification (intermediate or high [43], Fisher’s exact test). *p-*values are indicated in the figures.

Upon analysis, we excluded a number of samples because there were no GMNN-positive cells detected (n = 6) or no visible RAD51-foci in cells that did not undergo hyperthermia treatment (n = 5). In the remaining samples (n = 37), we distinguished two categories of RAD51 focus patterns upon hyperthermia: (1) foci became smaller and dispersed or, (2) foci morphology appeared to be unaltered (Fig 4A). Tumors which exhibited a clear response towards hyperthermia, visible as small and dispersed RAD51-foci, represented the majority of the samples (64%, Fig 4B). We subdivided the frequency distribution based on technical preparation of the samples (undissociated or dissociated) and on the manner of damage-induction, but did not find statistically significant differences in distribution of RAD51-focus within these categories (Fig 4C). Moreover, two tumors that were subjected to the assay in both undissociated and dissociated format could be analyzed, and in these cases the individual experiments were qualified within the same RAD51-category. Furthermore, a frequency distribution subdivision based on clinical parameters available for 34 tumors revealed that the reaction of RAD51-foci upon hyperthermia was not dependent on tumor grade, stage, recurrence or *European Association of Urology* risk stratification [43] (Fig 4D).

## Discussion

In this work, we explored two *ex vivo* assays that are directed towards determining the effects of hyperthermia on urothelial cancer cells. For both assays, bladder tumor tissue or dissociated tumor cells were treated with hyperthermia *ex vivo* and the effects were measured in a short period afterwards. Specifically, we studied the effect of hyperthermia on induction of apoptosis by quantitating cells displaying cytochrome C release and on HR DNA damage repair capacity by qualitative and quantitative analysis of RAD51 foci.

Cytochrome C-release is an early step in the apoptosis response, which makes it an ideal candidate to measure the fraction of apoptotic cells at an early stage after certain treatments [38]. Therefore, we tested the cytochrome C-release assay to determine sensitivity of bladder tumors towards cisplatin and MMC, and to determine if hyperthermia adds to the cell killing potential of these therapies. We started with models of bladder cancer, the bladder cancer cell lines RT112 and T24, and find that cell killing in the cytochrome C-release corresponds to the survival fraction in colony survival. We then were able to perform this assay successfully in 8 out of 13 fresh tumors, and within this set different responses to specific therapeutic combinations are found. Moreover, the assay detects enhanced apoptosis by addition of hyperthermia in bladder cells, and could therefore be useful to predict treatment response *ex vivo*.

The enhanced apoptosis response found when hyperthermia is combined with cisplatin and MMC in bladder cancer can be due to one or more of the many modes of action that might enhance sensitivity towards cisplatin and MMC [18]. One of the effects which might be of particular importance and can easily be measured is hyperthermia-induced degradation of BRCA2, which alters RAD51-focus behavior and ultimately inhibits HR [19]. Studying RAD51-foci formed *ex vivo* has been used as a functional assay to determine the genetic HR-status of breast cancer tissue indirectly [28,29], and we therefore tested whether this assay can be used on bladder cells to determine the effects of hyperthermia on HR. We found that although hyperthermia induced BRCA2 degradation in both cell lines, there was a distinct difference in RAD51-focus appearance upon hyperthermia. In RT112 cells, foci became small and dispersed, while in T24 cells the number of foci decreased dramatically, but the morphology of the remaining RAD51 foci remained mainly intact. Both changes in RAD51-focus formation and morphology have been observed earlier, albeit at different temperatures [33]. This indicates that both a decrease in focus number as well as a change in morphology can be considered as a positive signal in the context of hyperthermia-effectivity. Interestingly, the small and dispersed foci in RT112 cells are also found when HeLa-cells were treated with temperatures exceeding 43°C. This type of foci has been suggested to form independent of BRCA2, and might be a result of replication stress [33,44]. This hypothesis is supported by the large number of spontaneous RAD51-foci in RT112 cells, indicating high levels of endogenous DNA breaks.

Next, we applied the RAD51 focus formation assay to 48 freshly isolated bladder tumor samples. Interestingly, five samples had no visible RAD51-foci prior to hyperthermia, suggesting that ~10% of the bladder tumors are inherently HR-deficient, a percentage similar to that previously reported for primary breast cancers [28]. Thus, in bladder cancer, this *ex vivo* assay can potentially be used to identify a group of patients that might benefit from precision anti-cancer treatments directed against HR-defective tumors (*i*.*e*. PARP-1 inhibition) [45]. An additional six tumor samples were excluded for technical reasons.

In the majority of the 37 samples that we could analyze (64%), we found a clear response after hyperthermia: induction of small and dispersed RAD51 foci. In the remainder of the samples, hyperthermia did not alter the morphology of the RAD51-foci. Unfortunately, factors like cell heterogeneity, a small number of GMNN positive cells and the possibility that a paraffin section did not contain an entire nucleus, restrict analysis of the outcomes of extensive quantification and qualification as performed in the cell lines. Because we cannot measure the number of RAD51 foci accurately, the group where RAD51 foci had similar morphology before and after hyperthermia can potentially contain samples where HR-deficiency was induced and where samples were non-responsive towards hyperthermia. It is likely that a number of the samples with morphologically similar foci before and after hyperthermia is indeed refractory to the effects of heat, as is illustrated by combining the RAD51-focus results with thermal enhancement ratios (TERs) calculated based on the results from the cytochrome C-release assay (B147 and B154, Table 1).

**Table 1:** RAD51 focus response and TER.

Tumor	RAD51 focus response	TER MMC	TER cisplatin
**B135**	n.d.	2.1	5.0
**B141**	Small/Dispersed	0.61	5.0
**B142**	Similar	2.0	1.2
**B143**	n.d.	1.5	1.9
**B147**	Similar	n.d.	n.s.
**B149**	Small/Dispersed	1.4	1.3
**B150**	N.D. (small Foci before HT)	1.1	1.4
**B154**	Similar	1.3	n.s.

TER = Thermal Enhancement Ratio. Calculated using the percentages of apoptotic cells treated with indicated chemotherapeutic detected in the Cytochrome C-assay. Percentage of apoptotic cells found with hyperthermia divided by that found at normal temperature. n.d. = not determined. n.s. = not significant, therefore no TER calculated

Using a small number of bladder tumors, our study provides a basis for the use of two *ex vivo* assays to classify the response of bladder tumors upon hyperthermia or chemotherapy. The cytochrome C-release assay could potentially be useful to predict the response of a tumor to a chemotherapeutic, and to predict whether hyperthermia might add to this treatment. Importantly, we are able to perform these treatments on samples cultured *ex vivo*, which would allow us to assess treatment response on a biopsy, and thus prior to administrating the treatment to the patient. We also demonstrated that the RAD51-focus formation assay can be used as a marker for hyperthermia-mediated induction of HR-deficiency in bladder cancer specimens, and that the RAD51-focus response could potentially be a biomarker for hyperthermia-efficacy. Although these first and small-scale tests indicate that both assays are feasible for bladder tumors, a larger follow-up study that matches patient data to investigated tumors is needed to determine the clinical relevance of the results.

## Supporting information

S1 Table An overview of the collected material. This table provides an overview of all collected tumor material.
(DOCX)

S2 Table Exact *p-*values belonging to Fig 1. This table provides an overview of the exact *p*-values belonging to Fig 1.
(DOCX)

S3 Table Exact *p-*values belonging to Fig 2. This table provides an overview of the exact *p*-values belonging to Fig 2.
(DOCX)

## References

[1] Steliarova-Foucher E, O’Callaghan M, Ferlay J, Masuyer E, Rosso S, Forman D, et al The European Cancer Observatory: A new data resource. Eur J Cancer. 2014;51(9):1131–43. 10.1016/j.ejca.2014.01.027 24569102 10.1016/j.ejca.2014.01.027

[2] Ploeg M, Aben KKH, Kiemeney LA. The present and future burden of urinary bladder cancer in the world. World J Urol. 2009 6;27(3):289–93. 10.1007/s00345-009-0383-3 19219610 10.1007/s00345-009-0383-3 PMC2694323

[3] Burger M, Catto JWF, Dalbagni G, Grossman HB, Herr H, Karakiewicz P, et al Epidemiology and risk factors of urothelial bladder cancer. Eur Urol. 2013;63(2):234–41. 10.1016/j.eururo.2012.07.033 22877502 10.1016/j.eururo.2012.07.033

[4] Ploussard G, Shariat SF, Dragomir A, Kluth LA, Xylinas E, Masson-Lecomte A, et al Conditional survival after radical cystectomy for bladder cancer: evidence for a patient changing risk profile over time. Eur Urol. 2014 8;66(2):361–70. 10.1016/j.eururo.2013.09.050 24139235 10.1016/j.eururo.2013.09.050

[5] Abdollah F, Gandaglia G, Thuret R, Schmitges J, Tian Z, Jeldres C, et al Incidence, survival and mortality rates of stage-specific bladder cancer in United States: a trend analysis. Cancer Epidemiol. 2013 6;37(3):219–25. 10.1016/j.canep.2013.02.002 23485480 10.1016/j.canep.2013.02.002

[6] Ploussard G, Daneshmand S, Efstathiou JA, Herr HW, James ND, Rodel CM, et al Critical analysis of bladder sparing with trimodal therapy in muscle-invasive bladder cancer: a systematic review. Eur Urol. 2014 7;66(1):120–37. 10.1016/j.eururo.2014.02.038 24613684 10.1016/j.eururo.2014.02.038

[7] van Kessel KEM, Zuiverloon TCM, Alberts AR, Boormans JL, Zwarthoff EC. Targeted therapies in bladder cancer: an overview of in vivo research. Nat Rev Urol. 2015 12;12(12):681–94. 10.1038/nrurol.2015.231 26390971 10.1038/nrurol.2015.231

[8] Longo TA, Gopalakrishna A, Tsivian M, Van Noord M, Rasch CR, Inman BA, et al A systematic review of regional hyperthermia therapy in bladder cancer. Int J Hyperth. 2016 6;32(4):381–9. 10.3109/02656736.2016.1157903 PMC5440174 27134130

[9] Volpe A, Racioppi M, D’Agostino D, Cappa E, Filianoti A, Bassi PF. Mitomycin C for the treatment of bladder cancer. Minerva Urol Nefrol. 2010 6;62(2):133–44. 20562793

[10] Issels RD. Hyperthermia adds to chemotherapy. Eur J Cancer. 2008;44(17):2546–54. 10.1016/j.ejca.2008.07.038 18789678 10.1016/j.ejca.2008.07.038

[11] Lammers RJM, Witjes JA, Inman BA, Leibovitch I, Laufer M, Nativ O, et al The role of a combined regimen with intravesical chemotherapy and hyperthermia in the management of non-muscle-invasive bladder cancer: a systematic review. Eur Urol. 2011 7;60(1):81–93. 10.1016/j.eururo.2011.04.023 21531502 10.1016/j.eururo.2011.04.023

[12] van Valenberg H, Colombo R, Witjes F. Intravesical radiofrequency-induced hyperthermia combined with chemotherapy for non-muscle-invasive bladder cancer. Int J Hyperth Off J Eur Soc Hyperthermic Oncol North Am Hyperth Gr. 2016 6;32(4):351–62. 10.3109/02656736.2016.1140232 26905963

[13] Arends TJH, Nativ O, Maffezzini M, de Cobelli O, Canepa G, Verweij F, et al Results of a Randomised Controlled Trial Comparing Intravesical Chemohyperthermia with Mitomycin C Versus Bacillus Calmette-Guerin for Adjuvant Treatment of Patients with Intermediate- and High-risk Non-Muscle-invasive Bladder Cancer. Eur Urol. 2016 6;69(6):1046–52. 10.1016/j.eururo.2016.01.006 26803476 10.1016/j.eururo.2016.01.006

[14] Dewhirst MW, Lee C-T, Ashcraft KA. The future of biology in driving the field of hyperthermia. Int J Hyperth. 2016;32(1):4–13. 10.3109/02656736.2015.1091093 26850697

[15] van den Tempel N, Horsman MR, Kanaar R. Improving efficacy of hyperthermia in oncology by exploiting biological mechanisms. Int J Hyperth. 2016 6;32(4):446–54. 10.3109/02656736.2016.1157216 27086587

[16] Alvarez-Berrios MP, Castillo A, Mendez J, Soto O, Rinaldi C, Torres-Lugo M. Hyperthermic potentiation of cisplatin by magnetic nanoparticle heaters is correlated with an increase in cell membrane fluidity. Int J Nanomedicine. 2013;8:1003–13. 10.2147/IJN.S38842 23493492 10.2147/IJN.S38842 PMC3593770

[17] Landon CD, Benjamin SE, Ashcraft KA, Dewhirst MW. A role for the copper transporter Ctr1 in the synergistic interaction between hyperthermia and cisplatin treatment. Int J Hyperth. 2013;29(6):528–38. 10.3109/02656736.2013.790563 PMC3895488 23879689

[18] van der Heijden AG, Dewhirst MW. Effects of hyperthermia in neutralising mechanisms of drug resistance in non-muscle-invasive bladder cancer. Int J Hyperth. 2016 6;32(4):434–45. 10.3109/02656736.2016.1155761 27098923

[19] Krawczyk PM, Eppink B, Essers J, Stap J, Rodermond H, Odijk H, et al Mild hyperthermia inhibits homologous recombination, induces BRCA2 degradation, and sensitizes cancer cells to poly (ADP-ribose) polymerase-1 inhibition. Proc Natl Acad Sci U S A. 2011;108(24):9851–6. 10.1073/pnas.1101053108 21555554 10.1073/pnas.1101053108 PMC3116433

[20] Oei AL, Vriend LEM, Crezee J, Franken N a. P, Krawczyk PM. Effects of hyperthermia on DNA repair pathways: one treatment to inhibit them all. Radiat Oncol. 2015;10(1):165. 26245485 10.1186/s13014-015-0462-0 PMC4554295

[21] Knipscheer P, Raschle M, Smogorzewska A, Enoiu M, Ho TV, Scharer OD, et al The Fanconi anemia pathway promotes replication-dependent DNA interstrand cross-link repair. Science. 2009 12;326(5960):1698–701. 10.1126/science.1182372 19965384 10.1126/science.1182372 PMC2909596

[22] Long DT, Raschle M, Joukov V, Walter JC. Mechanism of RAD51-dependent DNA interstrand cross-link repair. Science. 2011 7;333(6038):84–7. 10.1126/science.1204258 21719678 10.1126/science.1204258 PMC4068331

[23] Semlow DR, Zhang J, Budzowska M, Drohat AC, Walter JC. Replication-Dependent Unhooking of DNA Interstrand Cross-Links by the NEIL3 Glycosylase. Cell. 2016 10;167(2):498–511.e14. 10.1016/j.cell.2016.09.008 27693351 10.1016/j.cell.2016.09.008 PMC5237264

[24] Yang Z, Nejad MI, Varela JG, Price NE, Wang Y, Gates KS. A role for the base excision repair enzyme NEIL3 in replication-dependent repair of interstrand DNA cross-links derived from psoralen and abasic sites. DNA Repair (Amst). 2017 4;52:1–11. 28262582 10.1016/j.dnarep.2017.02.011 PMC5424475

[25] Jasin M, Rothstein R. Repair of strand breaks by homologous recombination. Cold Spring Harb Perspect Biol. 2013;5(11):a012740 10.1101/cshperspect.a012740 24097900 10.1101/cshperspect.a012740 PMC3809576

[26] Moynahan ME, Pierce AJ, Jasin M. BRCA2 is required for homology-directed repair of chromosomal breaks. Mol Cell. 2001;7(2):263–72. 11239455 10.1016/s1097-2765(01)00174-5

[27] Naipal KAT, Raams A, Bruens ST, Brandsma I, Verkaik NS, Jaspers NGJ, et al Attenuated XPC expression is not associated with impaired DNA repair in bladder cancer. PLoS One. 2015;10(4):e0126029 10.1371/journal.pone.0126029 25927440 10.1371/journal.pone.0126029 PMC4416023

[28] Naipal KAT, Verkaik NS, Ameziane N, van Deurzen CHM, Ter Brugge P, Meijers M, et al Functional ex vivo assay to select homologous recombination-deficient breast tumors for PARP inhibitor treatment. Clin Cancer Res. 2014 9;20(18):4816–26. 10.1158/1078-0432.CCR-14-0571 24963051 10.1158/1078-0432.CCR-14-0571

[29] Mutter RW, Riaz N, Ng CKY, Delsite R, Piscuoglio S, Edelweiss M, et al Bi-allelic alterations in DNA repair genes underpin homologous recombination DNA repair defects in breast cancer. J Pathol. 2017 3; 10.1002/path.4890 PMC5516531 28299801

[30] Lowry OH, Rosebrough NJ, Farr AL, Randall RJ. Protein measurement with the Folin phenol reagent. J Biol Chem. 1951;193(1):265–75. 14907713

[31] Franken NAP, Rodermond HM, Stap J, Haveman J, van Bree C. Clonogenic assay of cells in vitro. Nat Protoc. 2006;1(5):2315–9. 10.1038/nprot.2006.339 17406473 10.1038/nprot.2006.339

[32] Schindelin J, Arganda-Carreras I, Frise E, Kaynig V, Longair M, Pietzsch T, et al Fiji: an open source platform for biological image analysis. Nat Methods. 2012;9(7):676–82. 10.1038/nmeth.2019 22743772 10.1038/nmeth.2019 PMC3855844

[33] van den Tempel N, Laffeber C, Odijk H, van Cappellen WA, van Rhoon GC, Franckena M, et al The effect of thermal dose on hyperthermia-mediated inhibition of DNA repair through homologous recombination. Oncotarget. 2017; 10.18632/oncotarget.17861 PMC5546504 28574821

[34] van der Heijden AG, Jansen CFJ, Verhaegh G, O’donnell M a, Schalken J a, Witjes JA. The effect of hyperthermia on mitomycin-C induced cytotoxicity in four human bladder cancer cell lines. Eur Urol [Internet]. 2004;46(5):670–4. Available from: http://www.ncbi.nlm.nih.gov/pubmed/15474281 10.1016/j.eururo.2004.06.009 15474281 10.1016/j.eururo.2004.06.009

[35] Heijkoop ST, van Doorn HC, Stalpers LJA, Boere IA, van der Velden J, Franckena M, et al Results of concurrent chemotherapy and hyperthermia in patients with recurrent cervical cancer after previous chemoradiation. Int J Hyperth. 2014 2;30(1):6–10. 10.3109/02656736.2013.844366 24156619

[36] Walker MC, Parris CN, Masters JR. Differential sensitivities of human testicular and bladder tumor cell lines to chemotherapeutic drugs. J Natl Cancer Inst. 1987 8;79(2):213–6. 3474453

[37] Bedford P, Walker MC, Sharma HL, Perera A, McAuliffe CA, Masters JR, et al Factors influencing the sensitivity of two human bladder carcinoma cell lines to cis-diamminedichloroplatinum(II). Chem Biol Interact. 1987 1;61(1):1–15. 3815585 10.1016/0009-2797(87)90015-9

[38] Goldstein JC, Waterhouse NJ, Juin P, Evan GI, Green DR. The coordinate release of cytochrome c during apoptosis is rapid, complete and kinetically invariant. Nat Cell Biol. 2000 3;2(3):156–62. 10.1038/35004029 10707086 10.1038/35004029

[39] Vaughn JP, Cirisano FD, Huper G, Berchuck A, Futreal PA, Marks JR, et al Cell cycle control of BRCA2. Cancer Res. 1996;56(20):4590–4. 8840967

[40] Karkoulis PK, Stravopodis DJ, Voutsinas GE. 17-DMAG induces heat shock protein 90 functional impairment in human bladder cancer cells: knocking down the hallmark traits of malignancy. Tumour Biol. 2016 5;37(5):6861–73. 10.1007/s13277-015-4544-2 26662567 10.1007/s13277-015-4544-2

[41] Tashiro S, Kotomura N, Shinohara A, Tanaka K, Ueda K, Kamada N. S phase specific formation of the human Rad51 protein nuclear foci in lymphocytes. Oncogene. 1996 5;12(10):2165–70. 8668342

[42] Alabert C, Bianco JN, Pasero P. Differential regulation of homologous recombination at DNA breaks and replication forks by the Mrc1 branch of the S-phase checkpoint. EMBO J. 2009 4;28(8):1131–41. 10.1038/emboj.2009.75 19322196 10.1038/emboj.2009.75 PMC2683710

[43] Babjuk M, Bohle A, Burger M, Capoun O, Cohen D, Comperat EM, et al EAU Guidelines on Non-Muscle-invasive Urothelial Carcinoma of the Bladder: Update 2016. Eur Urol. 2017 3;71(3):447–61. 10.1016/j.eururo.2016.05.041 27324428 10.1016/j.eururo.2016.05.041

[44] Tarsounas M, Davies D, West SC. BRCA2-dependent and independent formation of RAD51 nuclear foci. Oncogene. 2003;22(8):1115–23. 10.1038/sj.onc.1206263 12606939 10.1038/sj.onc.1206263

[45] O’Connor MJ. Targeting the DNA Damage Response in Cancer. Mol Cell. 2015 11;60(4):547–60. 10.1016/j.molcel.2015.10.040 26590714 10.1016/j.molcel.2015.10.040

