# Supplementary material for: Ex vivo assays to predict enhanced chemosensitization by hyperthermia in urothelial cancer of the bladder
Source: PLoS One. 2018 Dec 14;13(12):e0209101. doi: 10.1371/journal.pone.0209101 (PMC6294360; doi:10.1371/journal.pone.0209101)
Supplement: S1 Table — This table provides an overview of all collected tumor material. (DOCX) [file pone.0209101.s001.docx]

### Supplemental Table 1 – An overview of all collected material

| **Number** | **Tumor status** | **Grade** | **Histology** | **Size (mm)** | **Number of lesions** | **Carcinoma *in situ*** | **< 1-yr recidive** | **Primary/ Recurrence** | **NMIBC/ MIBC** | **NMIBC EAU Risk stratifi-cation^39^** | **Previous treatment** | **RAD51 foci induced by** | **RAD51 qualification** | |
| --- | --- | --- | --- | --- | --- | --- | --- | --- | --- | --- | --- | --- | --- | --- |
|  |  |  |  |  |  |  |  |  |  |  |  |  | **FFPE** | **after dissociation** |
| B064 | T1 | G3 | UCC | <30 | 1 | No | Unknown | Primary | NMIBC | High | No | 5 Gy |  | Small/Dispersed |
| B070 | Ta | G3 | UCC | >30 | >5 | No | Yes | Recurrence | NMIBC | High | No | 10 Gy |  | Small/Dispersed |
| B073 | Ta | G2 | UCC | >30 | >5 | No | Yes | Recurrence | NMIBC | High | No | 5 Gy | Small/Dispersed |  |
| B080 | T1 | G2 | UCC | <30 | 2 | No | No | Primary | NMIBC | High | No | 10 Gy |  | Unaltered |
| B082 | Ta | G2 | UCC | <30 | 3 | No | Yes | Recurrence | NMIBC | Intermediate | No | 10 Gy |  | Small/Dispersed |
| B083 | T1 | G3 | UCC | >30 | 2 | No | No | Recurrence | NMIBC | High | No | 10 Gy |  | Unaltered |
| B086 | Ta | G2 | UCC | <30 | >5 | No | Unknown | Primary | NMIBC | Intermediate | No | 10 Gy |  | Unaltered |
| B091 | Ta | G2 | UCC | >30 | 1 | No | Unknown | Primary | NMIBC | Intermediate | No | 10 Gy |  | Small foci before HT |
| B094 | Ta | G2 | UCC | >30 | 2 | No | Unknown | Primary | NMIBC | Intermediate | No | 10 Gy |  | Small/Dispersed |
| B095 | Ta | G2 | UCC | <30 | 1 | No | Unknown | Primary | NMIBC | Intermediate | No | 10 Gy |  | Small/Dispersed |
| B108 | T1 | G2 | UCC | >30 | 1 | No | No | Primary | NMIBC | High | No | 10 Gy |  | Small foci before HT |
| B110 | Ta | G2 | UCC | <30 | 1 | No | No | Primary | NMIBC | Intermediate | No | 10 Gy |  | Fixation artefact |
| B116 | Ta | G2 | UCC | <30 | 2 | No | No | Primary | NMIBC | Intermediate | No | 5 Gy |  | Small/Dispersed |
| B117 | * | * | UCC | <30 | 3 | No | No | Primary | NMIBC | Intermediate | No | 5 Gy | Small/Dispersed |  |
| B119 | T1 | G3 | UCC | >30 | 1 | No | Unknown | Primary | NMIBC | High | No | 5 Gy |  | No GMNN + |
| B120 | Ta | G2 | UCC | >30 | 1 | No | No | Recurrence | NMIBC | Intermediate | No | 5 Gy |  | Small/Dispersed |
| B128 | T2 | G3 | UCC | >30 | 1 | No | Unknown | Primary | MIBC | n.a. | No | 5 Gy | Small/Dispersed |  |
| B129 | T1 | G3 | UCC | <30 | 1 | Unknown | Unknown | Primary | NMIBC | High | No | 5 Gy | Unaltered |  |
| B130 | Ta | G1 | UCC | >30 | 6 | No | No | Recurrence | NMIBC | High | BCG and MMC | 5 Gy | Small/Dispersed |  |
| B131 | T1 | G3 | UCC | <30 | 2 | No | Unknown | Primary | NMIBC | High | No | 5 Gy | No RAD51 foci |  |
| B132 | Ta | G2 | UCC | <30 | 1 | No | Yes | Recurrence | NMIBC | Intermediate | BCG | 5 Gy | Small/Dispersed |  |
| B134 | Ta | G2 | UCC | <30 | 1 | Unknown | Unknown | Primary | NMIBC | Intermediate | No | 5 Gy | Unaltered |  |
| B135 | Ta | G1 | UCC | <30 | 1 | No | No | Primary | NMIBC | Low | No | 5 Gy |  | (N.D.) |
| B137 | Ta | G2 | UCC | <30 | 2 | No | No | Recurrence | NMIBC | Intermediate | MMC | 5 Gy | Small/Dispersed |  |
| B138 | * | * | * | * | * | * | * | * | * | * | * | 5 Gy | Small/Dispersed |  |
| **Number** | **Tumor status** | **Grade** | **Histology** | **Size (mm)** | **Number of lesions** | **Carcinoma *in situ*** | **< 1-yr recidive** | **Primary/ Recurrence** | **NMIBC/ MIBC** | **NMIBC EAU Risk stratifi-cation^39^** | **Previous treatment** | **RAD51 foci induced by** | **RAD51 qualification** | |
|  |  |  |  |  |  |  |  |  |  |  |  |  | **FFPE** | **after dissociation** |
| B139 | Ta | G2 | UCC | <30 | 1 | No | No | Primary | NMIBC | Intermediate | No | 5 Gy | No GMNN + |  |
| B140 | Ta | G2 | UCC | <30 | 2 | No | No | Primary | NMIBC | Intermediate | No | 5 Gy | Small/Dispersed |  |
| B141 | Ta | G2 | UCC | <30 | 1 | No | No | Primary | NMIBC | Intermediate | No | 5 Gy | Small/Dispersed |  |
| B142 | Ta | G2 | UCC | <30 | 4 | No | Yes | Recurrence | NMIBC | Intermediate | BCG | 5 Gy | Unaltered |  |
| B143 | Ta | G2 | UCC | <30 | 1 | No | No | Primary | NMIBC | Intermediate | No | 5 Gy | No GMNN + |  |
| B144 | T1 | G3 | UCC | >30 | 1 | Unknown | No | Primary | NMIBC | High | No | 5 Gy | Fixation artefact |  |
| B145 | T1 | G3 | UCC | <30 | 3 | Yes | No | Primary | NMIBC | High | No | 5 Gy | Small/Dispersed |  |
| B146 | Ta | G2 | UCC | >30 | 1 | No | Unknown | Primary | NMIBC | Intermediate | No | 5 Gy | Unaltered |  |
| B147 | Ta | G2 | UCC | <30 | 5 | No | Yes | Recurrence | NMIBC | Intermediate | No | 5 Gy | Unaltered |  |
| B148 | T2 | G3 | UCC | >30 | 1 | No | No | Primary | MIBC | n.a. | No | 5 Gy | No RAD51 foci |  |
| B149 | T2 | G3 | UCC | >30 | 1 | No | Yes | Recurrence | MIBC | n.a. | MMC | 5 Gy | Small/Dispersed |  |
| B150 | Ta | G2 | UCC | <30 | 1 | No | No | Primary | NMIBC | Intermediate | No | 5 Gy |  | Small foci before HT |
| B152 | T1 | G3 | UCC | >30 | 1 | Yes | No | Primary | NMIBC | High | No | 5 Gy | Unaltered |  |
| B153 | Ta | G2 | * | * | * | * | * | * | NMIBC | * | * | 5 Gy |  |  |
| B154 | * | * | * | * | * | * | * | * | * | * | * | 5 Gy | No GMNN + | Unaltered |
| B156 | Ta | G2 | UCC | <30 | 10 | No | Yes | Recurrence | NMIBC | Intermediate | MMC | Zeocin | Small/Dispersed | Small/Dispersed |
| B157 | T2 | G3 | UCC | >30 | 1 | Yes | No | Primary | MIBC | n.a. | No | Zeocin | Small/Dispersed |  |
| B158 | T1 | G3 | UCC | >30 | 1 | Unknown | No | Recurrence | NMIBC | High | Unknown | 5 Gy | Small/Dispersed |  |
| B159 | T2 | G3 | UCC | >30 | 1 | Yes | No | Primary | MIBC | n.a. | No | 5 Gy | Small/Dispersed |  |
| B160 | T2 | G3 | UCC | <30 | 2 | Yes | Unknown | Primary | NMIBC | High | TURB | 5 Gy | Small/Dispersed |  |
| B161 | T2 | G3 | UCC | >30 | 1 | No | No | Primary | MIBC | n.a. | No | 5 Gy | Unaltered | Unaltered |
| B164 | Ta | G3 | UCC | <30 | 1 | No | Unknown | Primary | NMIBC | High | No | 5 Gy | Small/dispersed |  |
| B165 | T2 | G3 | UCC | <30 | 1 | No | Unknown | Primary | MIBC | n.a. | No | 5 Gy | No GMNN + | No GMNN + |
| B167 | Ta | G2 | UCC | <30 | 3 | No | No | Recurrence | NMIBC | Intermediate | No | 5 Gy | Unaltered |  |
| B168 | Ta | G2 | UCC | <30 | 3 | No | No | Recurrence | NMIBC | Intermediate | No | 5 Gy | Unaltered |  |
| B169 | Ta | G2 | UCC | <30 | 2 | No | Yes | Recurrence | NMIBC | Intermediate | No | 5 Gy | No GMNN + | No GMNN + |

* Sample contained tumor cells and was therefore included in analyses. However, indicated data could not be retrieved and samples was therefore omitted from relevant subgroup analysis in Fig 4B

n.a. Not applicable

n.d. Not determined
