# Supplementary material for: Ex vivo assays to predict enhanced chemosensitization by hyperthermia in urothelial cancer of the bladder
Source: PLoS One. 2018 Dec 14;13(12):e0209101. doi: 10.1371/journal.pone.0209101 (PMC6294360; doi:10.1371/journal.pone.0209101)
Supplement: S2 Table — This table provides an overview of the exact p-values belonging to Fig 1. (DOCX) [file pone.0209101.s002.docx]

### Supplemental Table 2 –Exact *p*-values belonging to Figure 1

| **Panel** | **Cell line** | **Condition** | ***p*-value** |
| --- | --- | --- | --- |
| D | RT112 | 20 µg/mL MMC - 37 °C vs 42 °C | 1.908 · 10^-9^ |
|  |  | 40 µg/mL MMC - 37 °C vs 42 °C | 1.017 · 10^-3^ |
|  |  | 60 µg/mL MMC - 37 °C vs 42 °C | 0.1138 |
|  | T24 | 20 µg/mL MMC - 37 °C vs 42 °C | 5.917 · 10^-149^ |
|  |  | 40 µg/mL MMC - 37 °C vs 42 °C | 1.337 · 10^-80^ |
|  |  | 60 µg/mL MMC - 37 °C vs 42 °C | 0.1935 |
| E | RT112 | 5 µg/mL Cispt - 37 °C vs 42 °C | 0.07525 |
|  |  | 20 µg/mL Cispt - 37 °C vs 42 °C | 6.751 · 10^-15^ |
|  |  | 40 µg/mL Cispt - 37 °C vs 42 °C | 7.121 · 10^-9^ |
|  | T24 | 5 µg/mL Cispt - 37 °C vs 42 °C | 8.009 · 10^-83^ |
|  |  | 20 µg/mL Cispt - 37 °C vs 42 °C | 2.364 · 10^-13^ |
|  |  | 40 µg/mL Cispt - 37 °C vs 42 °C | 5.350 · 10^-56^ |
