# Supplementary material for: Ex vivo assays to predict enhanced chemosensitization by hyperthermia in urothelial cancer of the bladder
Source: PLoS One. 2018 Dec 14;13(12):e0209101. doi: 10.1371/journal.pone.0209101 (PMC6294360; doi:10.1371/journal.pone.0209101)
Supplement: S3 Table — This table provides an overview of the exact p-values belonging to Fig 2. (DOCX) [file pone.0209101.s003.docx]

### Supplemental Table 3 – Exact *p*-values belonging to Figure 2

| **Panel** | **Tumor** | **Condition** | ***p*-value** |
| --- | --- | --- | --- |
| A | B135 | Ctrl 37 °C vs MMC 37 °C | 1.7595 · 10^-118^ |
|  |  | Ctrl 37 °C vs Cispt 37 °C | 7.790 · 10^-136^ |
|  |  | MMC - 37 °C vs 42 °C | 7.506 · 10^-103^ |
|  |  | Cispt - 37 °C vs 42 °C | 10^-∞^ |
| B | B141 | MMC - 37 °C vs 42 °C | 5.155 · 10^-13^ |
|  |  | Cispt - 37 °C vs 42 °C | 2.987 · 10^-19^ |
| C | B142 | Ctrl 37 °C vs MMC 37 °C | 5.934 · 10^-40^ |
|  |  | Ctrl 37 °C vs Cispt 37 °C | 1.219 · 10^-70^ |
|  |  | MMC - 37 °C vs 42 °C | 2.021 · 10^-32^ |
|  |  | Cispt - 37 °C vs 42 °C | 3.993 · 10^-6^ |
| D | B143 | Ctrl 37 °C vs MMC 37 °C | 1.817 · 10^-4^ |
|  |  | Ctrl 37 °C vs Cispt 37 °C | 1.014 · 10^-8^ |
|  |  | MMC - 37 °C vs 42 °C | 0.01666 |
|  |  | Cispt - 37 °C vs 42 °C | 2.058 · 10^-5^ |
| E | B147 | Cispt - 37 °C vs 42 °C | 0.4963 |
| F | B149 | MMC - 37 °C vs 42 °C | 1.791 · 10^-4^ |
|  |  | Cispt - 37 °C vs 42 °C | 8.246 · 10^-3^ |
| G | B150 | Ctrl 37 °C vs MMC 37 °C | 0.2171 |
|  |  | Ctrl 37 °C vs Cispt 37 °C | 4.640 · 10^-9^ |
|  |  | MMC - 37 °C vs 42 °C | 0.03792 |
|  |  | Cispt - 37 °C vs 42 °C | 1.951 · 10^-3^ |
| H | B154 | MMC - 37 °C vs 42 °C | 1.781 · 10^-4^ |
|  |  | Cispt - 37 °C vs 42 °C | 0.08146 |
